# Azithromycin and Doxycycline Resistance Profiles of U.S. Mycoplasma genitalium Strains and Their Association with Treatment Outcomes

**DOI:** 10.1128/JCM.00819-21

**Published:** 2021-10-19

**Authors:** Gwendolyn E. Wood, Nicole L. Jensen, Sabina Astete, Jørgen S. Jensen, George E. Kenny, Christine M. Khosropour, Catherine W. Gillespie, Lisa E. Manhart, Patricia A. Totten

**Affiliations:** a Department of Medicine, University of Washington, Seattle, Washington, USA; b Statens Serum Institut, Copenhagen, Denmark; c Department of Epidemiology, University of Washington, Seattle, Washington, USA; d Center for AIDS and STD, University of Washington, Seattle, Washington, USA; e Department of Global Health, University of Washington, Seattle, Washington, USA; Marquette University

**Keywords:** azithromycin, doxycycline, antibiotics, MICs, *Mycoplasma genitalium*, antibiotic resistance, antimicrobial agents, nongonococcal urethritis

## Abstract

Mycoplasma genitalium is a sexually transmitted bacterium associated with nongonococcal urethritis (NGU) in men and cervicitis, endometritis, and pelvic inflammatory disease in women. Effective treatment is challenging due to the inherent, and increasingly acquired, antibiotic resistance in this pathogen. In our treatment trial conducted from 2007 to 2011 in Seattle, WA, we demonstrated poor efficacy of azithromycin (AZM) and doxycycline (DOX) against M. genitalium among men with NGU. In the present study, we cultured M. genitalium from 74 of 80 (92.5%) PCR-positive men at enrollment (V-1) and defined the MICs of AZM (*N* = 56 isolates) of DOX (*N* = 62 isolates). Susceptibility to AZM was bimodal; MICs were >8 μg/ml (44.6%) and <0.004 μg/ml (55.4%) for these isolates. The association of MIC with treatment efficacy was determined for men initially treated with either AZM (*N* = 30) or DOX (*N* = 24). Men treated with AZM were more likely to experience microbiologic treatment failure (*P* < 0.001) if infected with isolates that had AZM MICs of >8 μg/ml (18/18 men) than those with isolates that had AZM MICs of <0.004 μg/ml (1/12 men). Clinical treatment failure also was more likely to occur (*P* = 0.002) with AZM MICs of >8 μg/ml (12/18 men) than with AZM MICs of <0.004 μg/ml (1/12 men). In contrast, DOX MICs ranged from <0.125 to 2 μg/ml and were not correlated with microbiologic (*P* = 0.71) or clinical treatment (*P* = 0.41) failure, demonstrating no relationship between DOX MICs and treatment efficacy. Given the rapid spread of AZM resistance and the emergence of quinolone resistance, the current second-line therapy, monitoring MICs and evaluating other potential treatments for M. genitalium will be critical.

## INTRODUCTION

Mycoplasma genitalium is a reproductive tract pathogen for which culture is complicated and effective treatment regimens are challenging. This cell wall-less bacterium has been implicated in the etiology of nongonococcal urethritis (NGU) in men (1, 2) and cervicitis (3), endometritis (4), and pelvic inflammatory disease (5) in women (reviewed in references 6 and 7). Increasing evidence also associates M. genitalium with preterm birth, spontaneous abortion, and tubal factor infertility in women (reviewed in references 7 and 8). Like many other sexually transmitted pathogens, M. genitalium is also associated with increased risk of cervical shedding, transmission, and acquisition of HIV (9, 10). Although the prevalence of M. genitalium in a U.S. general population-based study of young adults appears low (1%), it was more than twice as common as Neisseria gonorrhoeae (11). Prevalence in sexual health clinics is substantially higher, ranging from 10% to 20% in several studies (12–14).

M. genitalium was first cultured in 1981 from urethral specimens of two men with urethritis by using complex growth medium formulated for a plant mycoplasma species (15). One of these strains, G37, was further characterized and designated the type strain for this species (16). The difficulty of culturing M. genitalium from clinical specimens precluded the characterization of additional strains until Jensen and Hamasuna et al. (17, 18) developed a Vero cell coculture technique in which growth is detected by an increase in genomes over time by quantitative PCR. Using these coculture methods, Hamasuna et al. (19) developed *in vitro* drug susceptibility assays comparing growth of M. genitalium in serial dilutions of antibiotics to growth in antibiotic-free medium after 21 to 28 days. These assays led to the detection of azithromycin (AZM)-resistant M. genitalium organisms, which were linked to single nucleotide polymorphisms (SNPs), termed macrolide resistance mutations (MRMs), in its single 23S rRNA gene (20). Unfortunately, only a few laboratories (17, 18) culture and perform MICs for M. genitalium, and no reports of the *in vitro* antibiotic susceptibilities of isolates in relation to clinical treatment outcomes have been published.

We conducted a randomized controlled trial comparing the efficacy of AZM and doxycycline (DOX) for treatment of NGU from 2007 to 2011 in Seattle, WA (21). M. genitalium was detected by PCR in urine of 13% of men with NGU, and AZM treatment resulted in clinical and microbiologic cure in only 63.2% and 39.5% of these men, respectively. DOX treatment efficacy was also low, with 48.1% of men cured clinically and 29.6% cured microbiologically. In the present study, we cultured the M. genitalium strains infecting these men, assessed diversity by strain typing, determined *in vitro* susceptibility to AZM and DOX, and correlated these MICs with treatment efficacy. This collection may be useful for future studies assessing the efficacy of other potential treatments for this reproductive tract pathogen.

## MATERIALS AND METHODS

### Overview of the treatment trial.

The population evaluated in our randomized clinical trial of the effectiveness of AZM versus DOX for treatment of NGU has been described (21). In this parent study, men with urethritis (visible urethral discharge or ≥5 polymorphonuclear leukocytes [PMNs]/×1,000 magnification microscopic field in Gram-stained smear) were enrolled at the Public Health-Seattle and King County STD clinic from 2007 to 2011. Men with Neisseria gonorrhoeae detected by Gram stain were excluded. At the enrollment visit (V-1), men were randomized to treatment with AZM (1 g single dose) or DOX (100 mg twice daily for 7 days) and asked to return 3 weeks later. When the men returned for their second visit (V-2), they were assessed for clinical cure (<5 PMNs/×1,000 magnification Gram-stained smear and no urethral discharge) and microbiologic cure (absence of genital pathogens). Of the 80 M. genitalium-positive men enrolled, 44 were treated with AZM and 36 were treated with DOX at V-1. At V-2, four and seven men were lost to follow-up in the AZM and DOX treatment groups, respectively, leaving a total of 69 men at V-2.

All study procedures were approved by the University of Washington Human Subjects Division. Laboratory personnel were blinded to the clinical status of enrolled patients.

### Culture of M. genitalium strains from clinical specimens.

Urine from men enrolled in the trial were processed within 16 h of collection to maintain the viability of M. genitalium. Urine (2 ml) was centrifuged at 21,000 × *g* for 20 min at 4°C, resuspended in 400 μl of mycoplasma transport medium (22), and frozen at −80°C until M. genitalium PCR results were obtained. M. genitalium isolates were cultured using a modification of the Vero cell coculture technique developed by Hamasuna et al. (19). Briefly, 1 × 10^5^ Vero cells were plated in 25-cm^2^ tissue culture flasks in Eagles minimal essential medium (EMEM; ATCC) supplemented with 10% fetal bovine serum (FBS) and 100 U/ml penicillin. After overnight incubation at 37°C in 5% CO_2_, Vero cells were washed with phosphate-buffered saline (PBS), fresh medium was added (8.5 ml EMEM supplemented with 10% FBS, 6% yeast dialysate, penicillin [100 U/ml], colistin [30 μg/ml], polymyxin B [50 μg/ml]; antibiotics purchased from Sigma), and flasks were inoculated with 100 μl of thawed processed urine specimen. Flasks were incubated for 28 days, with collection of aliquots of culture supernatants each week for frozen stocks (1 ml) and to detect growth by M. genitalium-specific quantitative PCR (qPCR) (23). Additional Vero cells were not added during incubation. While optimizing these culture methods, we found that the source of FBS (manufacturer and lot number) was critical for optimal growth. Gibco heat-inactivated, Performance Plus FBS (catalog number [cat. no.] 10082-147) was used for these experiments. Some lots of FBS were unable to support the growth of M. genitalium; whether this was due to inherent differences among animals or possibly the presence of residual antibiotics in the sera is not known.

### Strain typing.

M. genitalium strain typing was performed as described in the study by Jensen et al. (24), which identifies SNPs in the 5′ region of the *mgpB* gene. Strain type sequences were compared to each other and to previously identified sequences (25–27). As laboratory contamination of patient cultures by the G37 type strain has been reported in other studies (see below), we performed strain typing on the processed urine and corresponding cultured isolate for 25 patients. In every case, the strain present in urine was identical to the cultured isolate, confirming that our workflow prevented cross contamination by other M. genitalium strains present in the laboratory.

### Antibiotic susceptibility testing.

**FIG 1:**
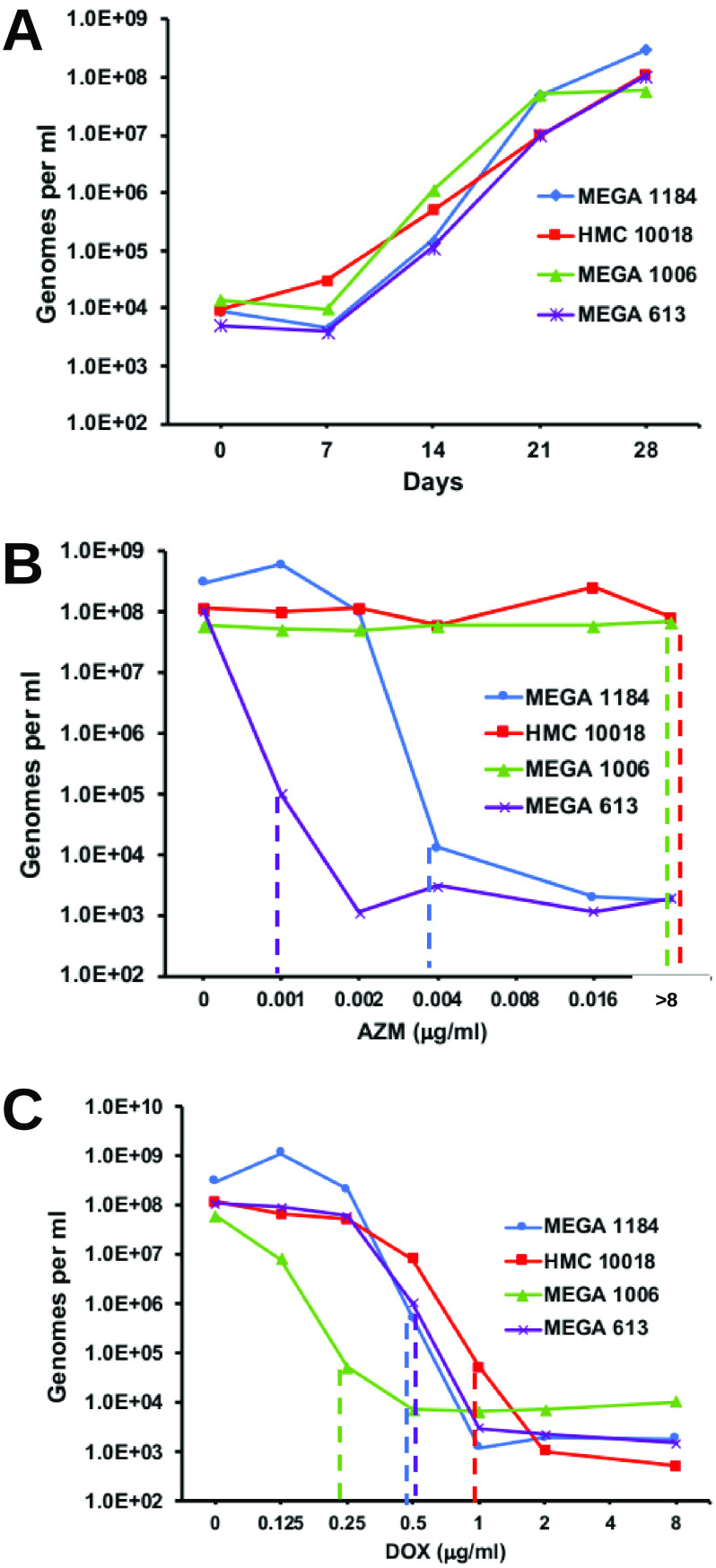
Detection of growth, AZM MICs, and DOX MICs for four isolates of M. genitalium (MEGA 1184, HMC 10018, MEGA 1006, and MEGA 613) in Vero cell coculture. (A) Growth without antibiotics is indicated by an increase in genomes, measured by M. genitalium-specific qPCR. Dotted lines indicate MICs, defined by 99% growth inhibition compared to no antibiotic, determined in serial 2-fold dilutions of AZM (B) or DOX (C) at 28 days postinoculation.

To determine the MICs of AZM and DOX for M. genitalium clinical isolates, we adapted the methods of Hamasuna et al. (19). Vero cells (4 × 10^3^ cells per well) were cultured overnight in 24-well tissue culture plates, using only the middle eight wells to avoid “edge effects” and avoid fungal contamination during incubation. Adherent Vero cells were washed twice with PBS, and then 1 ml of fresh medium (EMEM supplemented with 10% FBS and 6% yeast dialysate) containing 2 × 10^4^ genomes of M. genitalium and 1 ml of medium containing AZM (final concentrations of 0.001, 0.002, 0.004, and 8 μg/ml) or DOX (final concentrations of 0.125, 0.25, 0.5, 1.0, 2.0, 4.0, and 8.0 μg/ml) were added. Each antibiotic concentration was tested in a single well, and each well was compared to three control wells containing no antibiotic, consistent with previous reports (19, 28–30). As AZM was dissolved in ethanol, we included an additional control well containing 0.8% ethanol, corresponding to the highest concentration in the assays. No inhibition of M. genitalium growth was observed for this “ethanol-only” control. The plates were wrapped with parafilm to prevent evaporation and incubated for 28 days, with collection of aliquots (150 μl) weekly for DNA isolation (MasterPure complete DNA and RNA purification kit; Lucigen) and qPCR (described above). Triplicate wells containing no antibiotics were used to track the growth of M. genitalium over time. M. genitalium genomes at day 28 in antibiotic-treated wells were quantified in triplicates by qPCR and compared to a standard curve of known M. genitalium genome quantities (in quadruplicates). MICs were defined as the minimum concentration of antibiotic that inhibited growth by 99% compared to growth in control wells containing no antibiotic. Typical results are shown in Fig. 1.

### Detection of macrolide resistance-associated mutations in M. genitalium.

Sequences consistent with MRMs were detected by pyrosequencing of region V of the 23S rRNA gene as described previously (31) or by standard sequencing methods. Mutations were identified in base pairs 2058 and 2059 (Escherichia coli numbering).

### Statistical analyses.

The association of AZM MICs and AZM SNP types with microbiologic and clinical outcomes was calculated by Fisher’s exact test; the association of DOX MICs with these outcomes was evaluated by the Mann-Whitney U test.

### Data availability.

All new sequences have been deposited in GenBank under accession numbers MT594348 to MT594354 as indicated in the footnote of Table 1.

**TABLE 1:** Characteristics of M. genitalium strains detected at enrollment (visit 1) and response to treatment with AZM and DOX, detected at visit 2

Isolate no.^*a*^	Strain type^*b*^	Visit 1				Visit 2	
		MIC (μg/ml)		AZM SNP type^*c*^	Treatment	Clinical cure/failure^*d*^	M. genitalium PCR^*e*^
		DOX	AZM				
MEGA 223	J-6	0.25	<0.001	Wild type	AZM	Cure	Neg
MEGA 276	J-3	0.5	<0.001	Wild type	AZM	Cure	Neg
MEGA 1395	ND^*f*^	0.25	<0.001	Wild type	AZM	Cure	Neg
MEGA 76	UW-11	0.5	0.001	Wild type	AZM	Cure	Neg
MEGA 444	J-6	2	0.001	Wild type	AZM	Cure	Pos
MEGA 613	UW-3	0.5	0.001	Wild type	AZM	Failure	Neg
MEGA 1138	ND	0.5	0.001	Wild type	AZM	LTF	LTF
HMC 10014-1	ND	2	0.002	Wild type	AZM	Cure	Neg
MEGA 1404	GB-3	1	0.002	Wild type	AZM	Cure	Neg
MEGA 1423	GB-4	0.5	0.002	Wild type	AZM	LTF	LTF
MEGA 83	ND	1	0.004	Wild type	AZM	Cure	Neg
MEGA 285	J-7	1	0.004	Wild type	AZM	Cure	Neg
MEGA 430	J-3	0.5	0.004	Wild type	AZM	Cure	Neg
MEGA 520	UW-1	0.5	0.004	Wild type	AZM	Cure	Pos
HMC 10036-1	J-8	0.5	ND	Wild type	AZM	Failure	Pos
HMC 10052-1*	ND	ND	ND	Wild type	AZM	Cure	Neg
MEGA 218*	ND	ND	ND	Wild type	AZM	LTF	LTF
MEGA 709	ND	ND	ND	Wild type	AZM	Failure	Neg
MEGA 1299*	ND	ND	ND	Wild type	AZM	LTF	LTF
MEGA 1342	UW-10	ND	ND	Wild type	AZM	Cure	Neg
MEGA 1356*	J-3	ND	ND	Wild type	AZM	Cure	Neg
MEGA 1604*	ND	ND	ND	Wild type	AZM	Cure	Neg
MEGA 1656	ND	0.125	ND	Wild type	AZM	Cure	Neg
MEGA 1797	J-4	0.125	ND	Wild type	AZM	Cure	Pos
HMC 10008-1	J-3	0.5	>8	A2058G	AZM	Failure	Pos
MEGA 74	ND	0.25	>8	A2058G	AZM	Cure	Pos
MEGA 216	J-39	2	>8	A2058C	AZM	Failure	Pos
MEGA 316	J-3	0.5	>8	A2059G	AZM	Failure	Pos
MEGA 784	GB-1	0.5	>8	A2059G	AZM	Failure	Pos
MEGA 814	ND	0.5	>8	A2059G	AZM	Cure	Pos
MEGA 968	GB-6	<0.125	>8	A2058G	AZM	Failure	Pos
MEGA 1161	J-5	1	>8	A2058G	AZM	Failure	Pos
MEGA 1183	ND	ND	ND	A2059G	AZM	Cure	Pos
MEGA 1226	ND	2	>8	A2059G	AZM	Cure	Pos
MEGA 1256	GB-2	0.25	>8	A2058G	AZM	Failure	Pos
MEGA 1272	J-51	0.25	>8	A2059G	AZM	Failure	Pos
MEGA 1289	J-3	1	>8	A2059G	AZM	Failure	Pos
MEGA 1312	J-3	1	>8	A2059G	AZM	Cure	Pos
MEGA 1421	UW-6	1	>8	A2059G	AZM	Failure	Pos
MEGA 1432	J-51	ND	ND	A2059G	AZM	Cure	Pos
MEGA 1439	J-39	2	>8	A2058C	AZM	Cure	Pos
MEGA 1473	J-5	0.25	>8	A2058G	AZM	Failure	Pos
MEGA 1616	ND	0.25	>8	A2059G	AZM	Failure	Pos
MEGA 1491	J-4	0.25	>8	A2059G	AZM	Cure	Pos
MEGA 97	ND	<0.125	0.002	Wild type	DOX	LTF	LTF
MEGA 735	J-22	<0.125	<0.001	Wild type	DOX	Failure	Pos
MEGA 31	J-51	0.125	0.002	Wild type	DOX	Failure	Pos
MEGA 1649	ND	0.125	ND	Wild type	DOX	Failure	Pos
MEGA 1704	ND	0.125	ND	Wild type	DOX	Failure	Neg
MEGA 601	J-2	0.25	0.004	Wild type	DOX	Failure	Pos
MEGA 1303	J-2	0.25	<0.001	Wild type	DOX	Failure	Pos
MEGA 1385	ND	0.25	0.004	Wild type	DOX	LTF	LTF
MEGA 1822	ND	0.25	ND	A2059G	DOX	Cure	Neg
MEGA 206	ND	0.5	0.004	Wild type	DOX	LTF	LTF
MEGA 625	J-21	0.5	0.004	Wild type	DOX	Failure	Pos
MEGA 750	J-6	0.5	<0.001	Wild type	DOX	Cure	Neg
MEGA 837	ND	0.5	>8	A2059G	DOX	Cure	Neg
MEGA 943	ND	0.5	>8	A2059G	DOX	Cure	Pos
MEGA 1117	ND	0.5	>8	A2059G	DOX	Failure	Pos
MEGA 1160	J-5	0.5	<0.001	Wild type	DOX	Failure	Pos
MEGA 1166	J-3	0.5	0.004	Wild type	DOX	Cure	Neg
MEGA 1193	GB-1	0.5	>8	A2059G	DOX	Cure	Neg
MEGA 1199	J-2	0.5	>8	A2059G	DOX	Failure	Pos
MEGA 1345	J-3	0.5	>8	A2059G	DOX	Cure	Pos
MEGA 1568	ND	0.5	<0.001	Wild type	DOX	Failure	Pos
HMC 10018-1	J-3	1	>8	A2058G	DOX	Failure	Pos
MEGA 769	ND	1	0.001	Wild type	DOX	Cure	Neg
MEGA 1184	GB-5	1	0.004	Wild type	DOX	Failure	Pos
MEGA 1331	UW-5	1	0.002	Wild type	DOX	Cure	Pos
MEGA 1591	ND	1	0.002	Wild type	DOX	Failure	Pos
HMC 10032-1	J-4	2	<0.001	Wild type	DOX	Failure	Pos
HMC 10022-1	ND	ND	ND	Wild type	DOX	LTF	LTF
MEGA 372	UW-7	ND	ND	A2058G	DOX	Cure	Neg
MEGA 1221	J-4	ND	ND	A2058G	DOX	LTF	LTF
MEGA 1442	ND	ND	ND	Wild type	DOX	Cure	Pos
MEGA 1476	ND	ND	ND	A2058G	DOX	LTF	LTF
MEGA 1561	ND	ND	ND	Wild type	DOX	Cure	Pos
MEGA 1711	ND	ND	ND	A2058G	DOX	Cure	Pos
MEGA 1724	ND	ND	ND	A2059G	DOX	LTF	LTF
MEGA 1768*	ND	ND	ND	Wild type	DOX	Cure	Pos

a M. genitalium isolates were recovered from all PCR-positive patients except those marked with “*.”

b Strain types marked “J” were previously described (25), those marked “GB” were identical to sequences previously submitted to GenBank (accession numbers: GB-1, FJ750828.1; GB-2, KC445152.1; GB-3, FJ750829.1; GB-4, FJ750832.1; GB-5, EU131381.1; and GB-6, MK673442.1). UW strain types have not been previously described and were deposited in GenBank (accession numbers: UW-1, MT594348; UW-3, MT594349; UW-5, MT594350; UW-6, MT594351; UW-7, MT594352; UW-10, MT594353; UW-11, MT594354).

c Mutations base pairs 2058 and 2059 of the M. genitalium 23S rRNA gene (E. coli numbering).

d LTF, lost to follow up.

e Pos, positive; Neg, negative.

f ND, not done.

## RESULTS

### Growth and strain typing of M. genitalium recovered from PCR-positive specimens.

M. genitalium was cultured from 74 of 80 (92.5%) PCR-positive men at enrollment (V-1). The M. genitalium isolates at V-1, their strain types, antibiotic susceptibilities, and treatment outcomes at V-2 are shown in Table 1. At V-2, M. genitalium was cultured from 41 of 45 (91.1%) PCR-positive men.

M. genitalium strain typing, performed on the V-1 isolates from 46 men (45 from cultures and 1 directly from processed urine), identified 25 different strain types. Eleven strain types were described by Hjorth et al. (25), six were identical to previously reported sequences deposited in GenBank (26, 27), and seven were unique to this study (Table 1). Eighteen of the strain types were found in single patients, seven strain types were found in 2 to 4 patients, and one strain type (J-3) was found in 10 patients. Although the J-3 strain type was found in 10 men, these isolates differed in AZM susceptibility (4 were susceptible, 6 were resistant), suggesting that this strain developed resistance while circulating in our patient population during the study or that this typing method incompletely discriminates between strains (32). We also determined strain types for M. genitalium that persisted from V-1 to V-2 in 29 of these men. In all cases, the strain type detected at V-1 was identical to the type found at V-2, consistent with the persistence of a single strain in each man or reinfection from an untreated partner harboring the same strain.

### Antimicrobial susceptibility testing.

**FIG 2:**
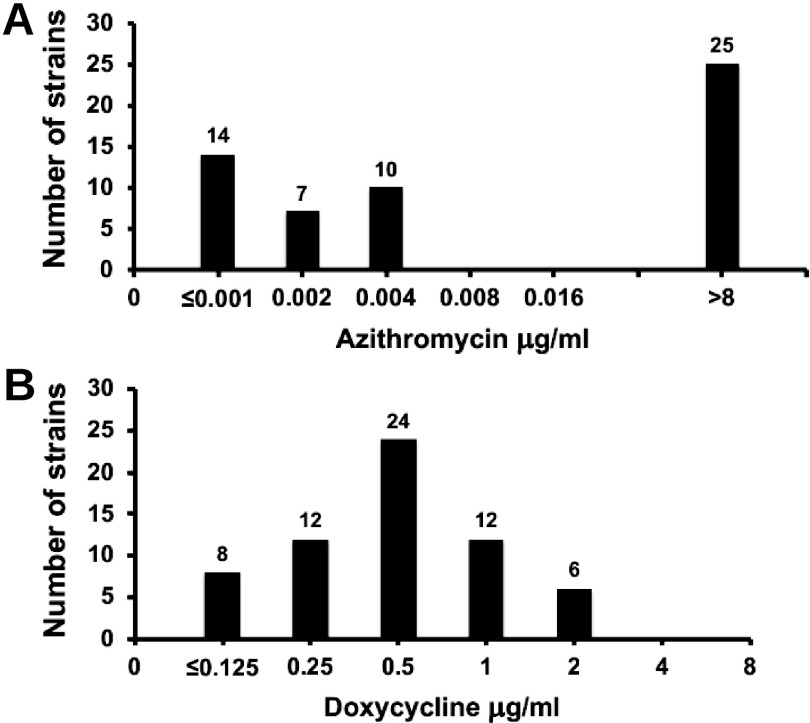
Distributions of the AZM (A) and DOX (B) MICs performed on 56 and 62 M. genitalium strains cultured at V-1, respectively.

We performed antimicrobial susceptibility tests for M. genitalium isolates obtained from men at enrollment (Fig. 2). The distribution of MICs of AZM was bimodal: 25 of 56 (44.6%) isolates had MICs of >8 μg/ml, and 31 (55.4%) had MICs of ≤0.004 μg/ml. In contrast, the DOX MICs for the 62 isolates tested formed a bell-shaped curve, with MIC_50_ and MIC_90_ values of 0.5 μg/ml and 2.0 μg/ml, respectively.

### AZM resistance genotyping.

AZM SNP typing of the 23S rRNA gene confirmed the MIC data: all 25 M. genitalium isolates with MICs of >8 μg/ml had MRMs associated with AZM resistance, and all 31 isolates with AZM MICs of ≤0.001 to 0.004 μg/ml had wild-type 23S rRNA sequences. Given this tight association between the presence of particular 23S rRNA SNPs and *in vitro* resistance in our study population, we performed AZM typing directly on the V-1 patient specimens from the remaining 24 men in our study. Altogether, we found that 33 (41.3%) of the 80 men enrolled in the study were infected with MRM-containing M. genitalium and 47 (58.8%) were infected with wild-type strains at enrollment. The SNPs detected in these MRM-containing isolates were A2059G (*N* = 20), A2058C (*N* = 2), and A2058G (*N* = 11) (see Table 1).

### Association of azithromycin resistance with clinical and microbiologic cure.

We determined the association between AZM MIC and microbiologic and clinical cure for 30 men who received AZM at enrollment and returned for evaluation at V-2 (Table 2). Of the 18 men infected with a strain with an MIC of >8 μg/ml, all remained M. genitalium PCR positive at V-2 compared to only 2 (16.7%) of the 12 men infected with a strain with an MIC of ≤0.004 μg/ml (*P* < 0.001). In addition, 12 (66.7%) of the men infected with a strain with an MIC of >8 μg/ml had persistent NGU at V-2 compared to 1 (8.3%) of the 12 men infected with a strain with an MIC of ≤0.004 μg/ml (*P* = 0.002). Together, these results demonstrate that the *in vitro* AZM resistance (>8 μg/ml) of the infecting strain at enrollment (V-1) was significantly associated with microbiologic and clinical failure after treatment (determined at V-2). We also assessed the association of MRMs with treatment failure among all men treated with AZM who returned for V-2 (Table 2). In this analysis, all 20 men infected with MRM-containing M. genitalium strains were PCR positive at V-2, while only 4 (20%) of 20 men infected with a wild-type strain remained PCR positive (*P* < 0.001). In addition, 12 (60%) of the 20 men infected with an MRM-containing strain were NGU positive at V-2 compared to 3 (15%) of 20 men infected with a wild-type strain (*P* = 0.008).

**TABLE 2:** Association of AZM MICs and AZM SNP types of M. genitalium strains from men treated with AZM at enrollment (V-1) with the microbiologic and clinical outcomes at their follow-up visit (V-2)

Outcome	AZM MICs at V-1^*a*^ (*N* = 30)			AZM SNPs at V-1^*b*^ (N = 40)		
	No. (%)		*P* value	No. (%)		*P* value
	>8 μg/ml	≤0.004 μg/ml		MRM^*c*^	Wild type	
Microbiologic outcome (V-2)						
PCR positive	18 (100)	2 (16.7)	<0.001	20 (100)	4 (20)	<0.001
PCR negative	0	10 (83.3)		0	16 (80)	
Clinical outcome (V-2)						
NGU	12 (66.7)	1 (8.3)	0.002	12 (60)	3 (15.0)	0.008
No NGU	6 (33.3)^*c*^	11 (91.6)		8 (40)	17 (85)	

a AZM MICs were performed on 32 cultures from men treated with AZM; 30 were evaluated for microbiologic and clinical cure at V-2 (two men were lost to follow-up; both were infected with AZM-sensitive strains at V-1).

b AZM SNP typing was performed on cultures or specimens from 44 men treated with AZM; 40 men were evaluated for microbiologic and clinical cure at V-2 (four were lost to follow-up; all four were infected with wild-type strains at V-1).

c MRMs were A2059G, A2058C, or A2048G (see Table 1).

### Characterization of azithromycin-sensitive isolates that persisted after treatment with AZM.

Of the 20 AZM-treated men infected with an AZM-susceptible strain (MIC ≤ 0.004 and/or wild-type 23S rRNA allele) at V-1 who returned for V-2, four (20%) were persistently infected with M. genitalium (Table 3). In each case, the V-2 isolate was AZM resistant as measured by MIC, presence of MRM, or both. We confirmed that the strain types of the M. genitalium isolates recovered from these four men were identical at V-1 and V-2, consistent with persistence of infection (or reinfection by an infected partner) and development of AZM resistance in the V-1 strain during treatment.

**TABLE 3:** Characteristics of isolates persisting after AZM treatment

Isolate	M. genitalium				
	Enrollment visit (V-1)^*a*^		V-2^*b*^		
	Strain type	MIC (μg/ml)	Strain type	MIC (μg/ml)	
HMC 10036	J-8	ND^*c*^	J-8	>8
MEGA 444	J-6	0.001	J-6	>8
MEGA 520	UW-1	0.004	UW-1	ND
MEGA 1797	J-4	ND	J-4	ND

a Wild-type AZM SNP.

b A2058G AZM SNP.

c ND, not done.

### Relationship between DOX MICs at V-1 and microbiologic and clinical outcomes at V-2.

DOX MICs, determined for M. genitalium strains cultured from 24 men treated with DOX at enrollment, ranged from <0.125 to 2 μg/ml for these V-1 strains (Table 4). However, there was no association between their DOX MICs and microbiologic (*P* = 0.71) and clinical (*P* = 0.41) failure at V-2. Of 12 DOX-treated men with DOX MICs evaluated at both V-1 and V-2, the MICs for the isolates from 11 men at these two time points were within one serial dilution of each other. An increased DOX MIC was detected in isolates from only one patient: 0.125 μg/ml at V-1 versus 1.0 μg/ml at V-2. In comparison, DOX MICs determined for V-1 and V-2 isolates obtained from 11 AZM-treated men were always within one serial dilution.

**TABLE 4:** Relationship between DOX MICs at V-1 and microbiologic (M. genitalium PCR^+^) and clinical failure (NGU^+^) at V-2^*a*^

MIC (μg/ml)	No. failed/total no. (%)	
	Microbiologic outcome	Clinical outcome
2	1/1 (100)	1/1 (100)
1	4/5 (80)	3/5 (60)
0.5	7/11 (63.6)	5/11 (45.5)
0.25	2/3 (66.7)	2/3 (66.7)
<0.125	3/4 (75)	4/4 (100)
Total	17/24 (70.8)	15/24 (62.5)

a DOX MICs were performed on cultures from 27 (75%) of the 36 men treated with DOX; 24 men were evaluated for microbiologic and clinical cure at V-1 (3 were lost to follow-up). There was no correlation between the DOX MIC values and microbiologic failure (*P* = 0.71) and clinical failure (*P* = 0.41). The median MIC values for all groups (microbiologic failure, microbiologic cure, clinical failure, and clinical cure) were 0.5 μg/ml (values of 0.125 μg/ml and <0.125 μg/ml were considered together as 0.125 μg/ml in this calculation).

## DISCUSSION

The association of M. genitalium with reproductive tract disease in men and women and its increasing antibiotic resistance worldwide highlights the need to monitor *in vitro* antibiotic susceptibilities and the relationship to treatment efficacy. Unfortunately, the fastidious nature and slow growth of M. genitalium has hampered the recovery of strains from clinical specimens in all but a few laboratories worldwide (17–20). To our knowledge, no such studies have been performed in the United States and very few U.S. strains have been isolated, limiting our knowledge of the geographic diversity of this pathogen. Herein, we report the culture, strain typing, antibiotic susceptibility testing, and assessment of AZM and DOX MIC values with clinical and microbiologic treatment outcomes for patients with NGU in Seattle, WA.

We cultured 74 M. genitalium isolates from PCR-positive urine specimens collected from 80 men at their enrollment visit (V-1) and from 67 PCR-positive men at subsequent visits. The recovered isolates were diverse: 26 different strain types were identified in a subset consisting of 46 men at enrollment. We confirmed the integrity of our culture methods by verifying that the strain type of M. genitalium isolates was identical to the strain type detected in the corresponding specimens in all of 25 men tested. This confirmation is important, as M. genitalium strain collections have been plagued with cross-contamination in the past. For example, the M. genitalium strains reportedly isolated from genital, respiratory, and synovial sites from 1985 to 1995 and deposited in ATCC (33, 34) are identical to the G37 type strain as determined by several methods (24, 35, 36), including the strain typing method used in our study (24) and whole-genome sequencing (37). More recently, Mondeja et al. (38) determined that cross-contamination between cultured isolates had occurred *in vitro* and that only 2 of 12 Cuban isolates could be considered new. As a precaution, we recommend that laboratories initiating the culture of M. genitalium from clinical sources confirm that the strain types of new isolates match those in the patient specimen.

We determined AZM MICs of the M. genitalium isolates cultured at enrollment to assess the relationship between MIC values and treatment outcomes. Similar to that to other studies (20, 39), the AZM MICs had a bimodal distribution (<0.001 to 0.004 or >8 μg/ml); 44.6% of isolates were resistant to >8 μg/ml. Thirty of the AZM-treated men infected with MIC-tested isolates at V-1 were assessed for treatment outcomes at V-2, revealing an association of >8 μg/ml MICs with microbiologic and clinical treatment failure (*P* < 0.001 and 0.002, respectively). Six (33%) of the 18 men infected with MRM-containing M. genitalium at V-1 lacked signs and symptoms of NGU at V-2, consistent with our previous report (40). The preponderance of high AZM MICs in our population and their correlation with AZM treatment failure were an early signal of declining efficacy of AZM in the United States. More recent data from Seattle, WA, demonstrates worsening local AZM resistance, reaching 62% of M. genitalium infecting men who have sex with women (40) and 90% in men who have sex with men (41). Similar increases in AZM resistance across the globe indicate that AZM is no longer the preferred treatment for NGU in many settings (42, 43) (http://www.sti.guidelines.org.au/sexually-transmissible-infections/mycoplasma-genitalium).

In our study, all isolates with high MICs (>8 μg/ml) contained previously described MRMs (39), and all isolates with low MICs (≤0.004 μg/ml) contained wild-type 23S rRNA alleles, allowing the detection of MRM as a surrogate for susceptibility to AZM, both of which were significantly associated with treatment failure. The development of PCR assays to detect MRMs in M. genitalium (44) shortly after the first discovery of macrolide-resistant strains in Australia in 2008 (20) has greatly facilitated the detection of macrolide-resistant M. genitalium (45). Macrolide resistance has become widespread, exceeding 50% in many locations (46–50), including the United States (51). The increased prevalence of AZM-resistant strains is due in part to the transmission of resistant strains. However, the ability of a single nucleotide change in the single genome copy of the 23S rRNA gene to confer resistance has played a large role. Most other bacteria have multiple 23S rRNA genes (e.g., Neisseria gonorrhoeae strains have 4 to 5 copies), attenuating macrolide resistance when only a few of their rRNA genes have this mutation (52). In our study, M. genitalium persisted in 4 of 20 men (20%) after AZM treatment despite demonstrated AZM susceptibility of these isolates at enrollment. The isolates cultured after treatment had AZM-resistant genotypes but unchanged strain types, consistent with the selection of *de novo* MRM mutations and/or the expansion of resistant variants present initially as a minor population. Similarly, Horner et al. (53) reported that resistance arose in 15% of AZM-treated patients. Cadosch et al. (54) calculated that 25%, 62%, and 84% of M. genitalium infections will resist AZM treatment, in France, Sweden, and Denmark, respectively, by 2025 if single-dose AZM continues as the default treatment. AZM resistance has already reached 100% in some study populations (48).

The low efficacy of DOX treatment in our study is consistent with previous randomized, double-blinded treatment trials in which >50% of men failed to clear M. genitalium (21, 55, 56), although the reasons for poor efficacy are not understood. Our study suggests that DOX treatment success is independent of the MIC values of the infecting strain, although the number of observations was small. Plasma DOX concentrations average 2 ± 1 μg/ml for a 100-mg oral dosage with a half-life of approximately 12 h (57). Although data regarding DOX levels in the male urethra are unknown, it is possible that the concentration of DOX at this site of infection is below that needed to completely eliminate M. genitalium, especially if drug doses are skipped and/or M. genitalium forms drug-resistant biofilms, as recently suggested (58). Interestingly, SNPs in the 16S rRNA gene of M. pneumoniae increased DOX MICs from 0.06 to 0.5 to 1.0 μg/ml (59). Furthermore, Berçot et al. (60) identified similar SNPs in the M. genitalium 16S rRNA gene in two patients after DOX treatment, and Le Roy et al. (61) found these mutations in M. genitalium-positive specimens from six patients, although these patients were not treated with DOX-tetracycline (TET), and *in vitro* MIC values were not obtained. Determining whether the M. genitalium strains in our study harbor these 16S rRNA SNPs might help determine if such mutations contribute to DOX treatment failure. In other organisms, including Mycoplasma hominis and *Ureaplasma* sp., high-level tetracycline resistance is conferred by the *tet*(M) gene, which is transmitted via an integrative conjugal element (62, 63). The discovery of conjugation in M. genitalium (64) raises the possibility that acquisition of *tet*(M) may further reduced DOX efficacy, although a constitutive promoter is apparently required for *tet*(M) expression in genetically engineered strains (65).

Although ineffective as a stand-alone treatment, DOX reduces organism load, a property that was exploited in resistance-guided treatment (66). In this strategy, patients were presumptively treated with DOX while awaiting detection of M. genitalium and MRM SNP typing using the ResistancePlus MG test (SpeeDx, Inc., Sydney, Australia). Patients infected with MRM-containing M. genitalium were treated with sitafloxacin (or moxifloxacin [MXF] in a subsequent study [67]), while those with the AZM-susceptible allele were treated with high dose azithromycin (2.5 g). This regimen increased microbiologic cure rates to 92.5%, even though prevalence of MRM was 62% in this patient population, and reduced the emergence of AZM resistance during treatment to 2.6% (66, 67). Accordingly, the UK (42) and Australian (http://www.sti.guidelines.org.au/sexually-transmissible-infections/mycoplasma-genitalium) guidelines recommend resistance-guided treatment strategies. In comparison, the 2016 European treatment guidelines for M. genitalium (43) recommend extended AZM (500 mg at day 1, followed by 250 mg at days 2 to 5) and MXF when MRMs are detected. Recognizing the alarming increase in treatment failures, in 2019, the CDC included M. genitalium on its Watch List of the Antibiotic Resistance Threats in the United States (https://www.cdc.gov/drugresistance/pdf/threats-report/2019-ar-threats-report-508.pdf). Currently, the 2015 U.S. CDC sexually transmitted disease (STD) treatment guidelines (68) indicate AZM (1 g single dose) or MXF for AZM-resistant infections (68), although updated treatment guidelines are forthcoming.

Unfortunately, MXF resistance is increasing, and there are few options to treat strains resistant to both MXF and AZM (28). An older tetracycline, minocycline (69, 70), has been effective in some cases of dual treatment failure. Lefamulin demonstrates promising *in vitro* activity (30), but its *in vivo* efficacy against M. genitalium has not yet been reported. Other drugs such as sitafloxacin (71) and pristinamycin (66, 70) are effective but are not available in the United States. Clearly, new treatment regimens are urgently needed to combat this increasingly resistant pathogen. *In vitro* MIC studies using clinical isolates such as those described in the present study are essential to evaluate these new drugs.
